# Editorial: Management of rare oncological cases

**DOI:** 10.3389/fonc.2025.1668671

**Published:** 2025-09-11

**Authors:** Lidia Castagneto-Gissey, Dragos Eugen Georgescu, Ferdinando Agresta

**Affiliations:** ^1^ Department of Surgery, Sapienza University of Rome, Rome, Italy; ^2^ Carol Davila University of Medicine and Pharmacy, Bucharest, Romania; ^3^ ULSS2 Marca Trevigiana, Treviso, Italy

**Keywords:** melanoma, sarcoma, neuroendocrine tumor, gynecologic cancer, molecular immunotherapy

The rapid evolution of oncology has brought new challenges and opportunities in the diagnosis and management of rare and complex tumors. Although infrequent in occurrence, these malignancies often carry poor prognoses due to diagnostic delays, limited treatment options, and lack of robust clinical trial data. The recent surge in detailed case reports, molecular analyses, and innovative treatment strategies contributes significantly to bridging these gaps. This editorial aims to synthesize recent findings from diverse rare tumor cases—spanning from gynecologic melanomas, to sarcomas, neuroendocrine neoplasms, and atypical metastases—and explore their implications for clinical practice and future research.

## Rare soft tissue and mesenchymal tumors

The occurrence of malignant granular cell tumors (GCTs) outside their usual head and neck locations, such as the chest wall, exemplifies diagnostic challenges posed by atypical presentations (Flauto et al.). GCTs, typically benign and slow growing, occasionally demonstrate aggressive behavior with recurrence and metastasis, necessitating repeated surgical interventions and careful long-term monitoring. Similarly, the pediatric case of pancreatic Ewing sarcoma described by Liu et al. expands our understanding of rare extraskeletal manifestations in an uncommon anatomical site, demonstrating the value of molecular diagnostics and tailored chemotherapeutic regimens in improving outcomes in aggressive pediatric malignancies.

Soft tissue sarcomas and stromal tumors constitute a heterogeneous group of mesenchymal neoplasms often posing diagnostic and therapeutic challenges. Bai et al. present uterine inflammatory myofibroblastic tumor (UIMT) involving the female reproductive tract with uncertain malignant potential. These lesions straddle the border between reactive and neoplastic processes and require excision for definitive diagnosis. The discovery of ALK gene rearrangements in UIMT has enabled targeted therapy with ALK inhibitors in select cases.

Gastrointestinal schwannomas, although generally considered benign peripheral nerve sheath tumors, occasionally present in unusual locations such as the colon. Yu et al. present a compelling case of a colonic schwannoma causing intestinal obstruction, an atypical clinical manifestation that underscores the importance of including mesenchymal tumors in the differential diagnosis of obstructive colorectal lesions.

The recurrence of benign tumors decades after surgery is another sobering reminder of the need for long-term surveillance. Lei et al. report a recurrent giant cystic lymphangioma of the peritoneum, which returned 20 years post-resection. The case raises important considerations regarding surveillance strategies and the latent potential for recurrence in ostensibly benign conditions.

## Rare cutaneous and skin-associated malignancies

Cutaneous neoplasms with mixed histopathologic features, such as the endocrine mucin-producing sweat gland carcinoma with mucinous carcinoma components reported by Sun et al., illustrate the complexity of tumor classification and metastatic potential that can alter clinical course significantly.

Zhang et al. explore the potential causal relationship between skin flora and cutaneous melanoma using Mendelian Randomization analyses, suggesting a novel area for further research in cancer etiology. Lambert-Swainston et al. describe a locally advanced basal cell carcinoma achieving pathologic complete response after combined neoadjuvant cemiplimab and vismodegib, paving the way for innovative immunotherapy-hedgehog inhibitor combinations.

Chen et al. conducted a population-based study on sebaceous carcinoma arising outside the head and neck, a subset that remains poorly characterized in the literature. Their findings reveal that while overall prognosis is favorable for non-head and neck sebaceous carcinoma, patients with distant metastases experience significantly poorer outcomes. Independent predictors of survival included age, tumor size, and stage, while undifferentiated tumors of the trunk demonstrated a higher propensity for distant spread. These insights underscore the potential value of stratified metastasis screening in high-risk subset.

The review by Pedersen et al. provides a comprehensive overview of Merkel cell carcinoma (MCC) biology, focusing on tumor immunogenicity and the tumor microenvironment’s role. MCC’s strong association with Merkel cell polyomavirus (MCPyV) and UV-induced mutations positions it as a prime candidate for immunotherapy. The introduction of immune checkpoint inhibitors (ICI), particularly PD-1 and PD-L1 inhibitors, has transformed MCC management. Clinical trials demonstrate durable responses in metastatic MCC, yet not all patients benefit equally. Resistance mechanisms, including immune escape and tumor heterogeneity, limit efficacy. Pedersen et al. emphasize ongoing efforts to identify biomarkers predictive of response, such as tumor mutational burden and MCPyV status. Additionally, combining ICI with radiotherapy is an area of interest, leveraging radiation-induced immunogenic cell death to augment immune responses. Ongoing trials evaluating combination regimens may further improve patient outcomes.

## Gynecologic and female reproductive tumors

The prognostic factors identified in uterine adenosarcoma through the SARCUT study subanalysis by Mancari et al. further refine risk stratification and guide postoperative treatment decisions in this rare uterine tumor.

Intravenous leiomyomatosis (IVL), a rare vascular smooth muscle tumor with potential for significant morbidity, is highlighted by Li et al. through the use of advanced ultrasound imaging techniques facilitating early diagnosis and successful surgical management. In parallel, Wang et al.’s case of urachal adenocarcinoma misdiagnosed as primary cervical adenocarcinoma draws attention to the critical need for accurate histopathological assessment in rare urologic malignancies and supports aggressive surgical interventions in advanced disease.

Feng et al.‘s report on primary retroperitoneal müllerian adenocarcinoma (PRMA) introduces an exceptionally rare tumor entity, emphasizing the role of immunohistochemistry in distinguishing this diagnosis from other retroperitoneal masses and the importance of considering PRMA in differential diagnoses. Additionally, Zan et al. describe a long-term progression-free survival case in advanced ovarian cancer treated with apatinib as first-line maintenance therapy, offering promising insights into cost-effective alternatives to standard maintenance regimens.

Zhang et al. provide valuable prognostic models delineating patient subgroups with high-grade neuroendocrine cervical cancer who derive survival benefits from radiotherapy, advancing personalized treatment approaches for this aggressive malignancy.

Wu et al. review a rare case of primary uterine non-Hodgkin’s lymphoma and provide valuable imaging-pathology correlation for diagnosis and treatment response assessment.

Liu et al. offer a comprehensive 13-year retrospective study on uterine smooth muscle tumors of uncertain malignant potential, revealing diagnostic difficulties and fertility implications, and highlighting myomectomy as a viable option in selected patients. Qing et al. described a synchronous presentation of cervical carcinoma *in situ* and Mullerian adenosarcoma diffusely involving the uterine cavity and cervix—an exceptionally rare co-occurrence. The diagnosis was established postoperatively, underscoring the diagnostic limitations posed by rare tumor types and the importance of histological reassessment. This case reinforces the imperative of considering multiple primary malignancies in gynecologic oncology, even in asymptomatic patients.

In a broader effort to characterize rare ovarian tumors, Chen et al. conduct a retrospective study of 40 cases of struma ovarii (SO), a rare ovarian teratoma composed predominantly of thyroid tissue. Their findings reveal that multimodal imaging—including ultrasound, contrast-enhanced ultrasound, and MRI—can significantly enhance diagnostic accuracy by identifying characteristic features such as cystic-solid components and distinctive signal patterns.

Uncommon gynecologic malignancies with unique histologies remain a diagnostic challenge. Zhang et al.’s report of primary cervical melanoma—a rare and aggressive neoplasm—underscores the importance of early detection and novel treatments like immunotherapy.

Zeng et al. detail a clear cell carcinoma of the cervix occurring without prior diethylstilbestrol exposure, contrasting with the classic epidemiology. This report highlights the necessity of considering rare histotypes in differential diagnoses and tailoring treatment accordingly. Such rare tumors often have limited data guiding management, emphasizing the value of case reports and registries to accumulate knowledge.

Detailed pathology, supported by immunohistochemistry and molecular techniques, provides critical diagnostic and prognostic information. Liang et al. explore the molecular profile of tubo-ovarian carcinosarcoma, revealing complex genomic alterations that may inform targeted therapies.

Dahl et al. introduce the use of patient-derived organoids (PDOs) from uterine carcinosarcomas to evaluate drug sensitivity *in vitro*. This platform allows personalized testing of chemotherapy and targeted agents, accelerating therapy optimization. PDOs represent a promising tool for rare cancers, enabling rapid functional assays that reflect tumor heterogeneity and microenvironmental factors, potentially transforming clinical decision-making.

Zhang et al. describe neuroendocrine cervical cancer, characterized by aggressive behavior and unique histology. Molecular markers such as synaptophysin and chromogranin assist diagnosis, while identification of somatostatin receptors opens therapeutic options. This case highlights benefits of combining chemotherapy, radiation, and immunotherapy in neuroendocrine cervical cancer. Given the poor prognosis and aggressive course, integrating multimodal therapy offers hope for improved outcomes. Immunotherapy’s expanding role across tumor types is evident from several reports, with checkpoint inhibitors inducing durable responses even in advanced disease. Radiotherapy’s combination with systemic therapies emerges as a key strategy, enhancing immunogenicity and response rates.

Many rare tumors mimic benign conditions on imaging, complicating diagnosis. Li et al. highlight uterine leiomyomatosis, which may resemble fibroids or sarcomas on ultrasound and MRI. Differentiating benign from malignant lesions is critical to prevent unnecessary aggressive treatment or delays.

Finally, fertility preservation in the treatment of rare peritoneal tumors is illustrated by Li et al. in a case of primary peritoneal serous borderline tumor (PPSBT). The patient underwent multiple fertility-sparing surgeries, affirming the feasibility of this approach in well-selected patients and supporting the value of multidisciplinary care and personalized surgical planning.

## Male genitourinary tumors

Huang et al. present a rare case of primary hepatoid adenocarcinoma of the lung in a patient with silicosis, demonstrating the aggressive nature of this tumor and the importance of early surgical intervention alongside emerging genetic testing approaches.

Furthermore, the report by Bai et al. on testicular choriocarcinoma with uncommon pelvic and pulmonary metastases highlights the aggressive clinical course in young males and the critical importance of early diagnosis and multidisciplinary care.

## Unusual metastatic patterns

The rare occurrence of metastatic deposits in unusual locations can mislead clinicians and delay appropriate management. Koh et al. report a case of scalp metastasis originating from breast carcinoma. Scalp lesions are often benign and rarely biopsied, underscoring the need for suspicion in patients with cancer history presenting with atypical cutaneous nodules.

Likewise, rare metastatic patterns, including renal cell carcinoma spreading to the thyroid (Xu et al.) or breast metastases originating from lung adenocarcinoma (Ding et al.), remind clinicians to maintain vigilance for atypical metastatic sites and emphasize the role of comprehensive diagnostic workups.

Hormonal receptor status variability in metastatic breast cancer to the colon, as reported by Li et al., adds an additional layer of complexity in treatment selection, underscoring the dynamic nature of tumor biology and the necessity for repeat biopsies in metastatic disease management.

## Rare gastrointestinal tumors

Therapeutic innovations and clinical management strategies are also a central theme. Fassi et al.’s report on regorafenib-associated erythrocytosis in metastatic extra-gastrointestinal stromal tumor (extra-GIST) presents a previously unrecognized adverse effect of a tyrosine kinase inhibitor, prompting closer hematologic monitoring during targeted therapy.

Carella et al. presented a rare case of primary solitary extraosseous plasmacytoma of the colon, mimicking a submucosal lesion. Notably, the lesion was detected during routine colonoscopy in a patient with minimal symptoms. Subsequent surgical and hematological workup confirmed the diagnosis. Given the tumor’s rarity and the paucity of definitive endoscopic features, this case highlights the role of comprehensive postoperative evaluation and interdisciplinary diagnostics.

Dong et al. reported three cases of rectal neuroendocrine tumors (NETs) incidentally diagnosed during anorectal surgeries for presumed benign conditions. Each case was managed surgically, with subsequent histopathologic confirmation revealing one G1 and two G2 rectal NETs. The absence of recurrence on follow-up reinforces the efficacy of early, complete excision. These findings support increased vigilance during routine proctologic procedures, particularly when atypical tissue is encountered.

Similarly, in the context of hereditary cancer syndromes, Sun et al. highlight a rare instance of gastric adenocarcinoma in a patient with Peutz–Jeghers syndrome, a genetic disorder more commonly associated with polyposis and increased risk for other malignancies. This case adds to the scarce but growing literature linking Peutz–Jeghers syndrome with gastric malignancy and raises questions about surveillance protocols in these patients.

## Pulmonary and thoracic rare cancers

Huang et al. present a rare case of primary hepatoid adenocarcinoma of the lung in a patient with silicosis, demonstrating the aggressive nature of this tumor and the importance of early surgical intervention alongside emerging genetic testing approaches.

## Immunotherapy and novel approaches

Uveal melanoma is the most common primary intraocular malignancy in adults but has limited treatment options when metastatic. Tran et al. present a case of metastatic uveal melanoma treated with combined radiotherapy and dual checkpoint blockade (nivolumab and relatlimab). The rationale involves radiation enhancing tumor antigen presentation and promoting T-cell infiltration, which checkpoint inhibitors can then amplify. Their report adds to emerging evidence supporting multimodal immunotherapy strategies in uveal melanoma. Despite historically poor responses to single-agent immunotherapy, combining checkpoint inhibitors with radiation may overcome immunosuppressive microenvironments. The observed durable partial response in this case underscores the potential of this approach and warrants further clinical investigation.

Several reports highlight the expanding role of immune checkpoint inhibitors in rare melanomas of mucosal and ocular origin. Gunenc et al. document effective use of combined relatlimab (anti-LAG3) and nivolumab (anti-PD-1) therapy in a conjunctival melanoma patient. These findings extend the success of immunotherapy beyond cutaneous melanoma, traditionally the most studied subtype. They emphasize the importance of molecular and immunologic profiling to identify candidates for checkpoint inhibition, particularly in tumors with low mutational burden but active immune microenvironments.

## Concluding perspective

The aggregation of rare tumor cases in centralized databases enables pattern recognition, prognostication, and development of evidence-based guidelines. Enhanced collaboration across institutions is essential to overcome the limitations posed by rarity. Molecular profiling and PDO models exemplify the move toward personalized oncology. Identifying actionable mutations and immune biomarkers will expand therapeutic options and improve survival in rare tumors. Many rare tumors lack standardized management protocols. Prospective trials, translational research, and integration of real-world data are crucial to define optimal care pathways.

These contributions underscore the importance of case reporting, precision diagnostics, and novel therapies. The diversity and complexity in these cases drive innovation and collaborative research in rare cancer management. A unified global strategy must ensure every rare case enlightens future guidelines, clinical decisions, and research priorities.

As we move forward, integrating advanced genomic profiling, immunotherapy, and real-world evidence from rare tumors is crucial. The work included in this Research Topic reminds us that every single report contributes meaningfully to the literature. These insights can inform trial design, guide biomarker discovery, and promote global collaboration.

The diverse and complex spectrum of rare tumors demands vigilant diagnosis, innovative therapies, and personalized approaches. Recent advances in immunotherapy, molecular diagnostics, and preclinical modeling herald a new era in oncology, turning clinical challenges into opportunities for improved patient care. Continuous reporting and collaboration remain vital to deepen understanding and refine management of these uncommon yet impactful malignancies.

Collectively, these cases illuminate the multifaceted challenges in diagnosing and treating rare tumors, from atypical presentations and rare metastatic patterns to evolving molecular diagnostics and novel therapeutic interventions. They underline the indispensable value of meticulous histopathological examination, advanced imaging modalities, and multidisciplinary management in optimizing patient outcomes.

As the medical community continues to encounter rare tumors with unique clinical behaviors, these reports contribute to a growing body of knowledge that will apprise future clinical guidelines and foster more precise, individualized care. The heterogeneity and rarity of these conditions demand ongoing research and international collaboration to unravel their complexities and improve prognostication and treatment paradigms.

We encourage more institutions to document, publish, and contribute to rare oncology registries. With comprehensive molecular diagnostics and multidisciplinary treatment, outcomes for patients with rare tumors can continue to improve. Each study included here contributes a valuable thread in the larger fabric of rare oncology knowledge and care.

